# Pharmacy Students’ Perception of E-Learning During the COVID-19 Pandemic Across the League of Arab States: A Regional Scoping Review

**DOI:** 10.3390/pharmacy14040099

**Published:** 2026-07-03

**Authors:** Haroon Malak, Madeeha Mirza, Stephen F. Gambescia, Basil H. Aboul-Enein

**Affiliations:** 1 Global Business Administration, Southern New Hampshire University, 2500 N River Road, Manchester, NH 03106, USA; 2 Pharmacy Department, Fairleigh Dickinson University, 285 Madison Avenue, Madison, NJ 07940, USA; 3 Health Administration, Drexel University, 3220 Market St., Philadelphia, PA 19104, USA; 4 Health & Society Program, College of Arts & Sciences, University of Massachusetts Dartmouth, 285 Old Westport Rd., North Dartmouth, MA 02747, USA; 5 Faculty of Public Health and Policy, London School of Hygiene & Tropical Medicine, 15–17 Tavistock Place, London WC1H 9SH, UK

**Keywords:** COVID-19, e-learning, pharmacy education, Arab world, higher education

## Abstract

The COVID-19 pandemic compelled higher education to resort to e-learning, posing new challenges to the teaching/learning of pharmacy students worldwide. While digital learning provided flexibility, diverse technological infrastructure and institutional availability of resources greatly influenced the student experience. This scoping review aims to assess the perceptions relating to the pivot to e-learning among pharmacy students in the League of Arab States due to the COVID-19 pandemic and how the shift affected student engagement, learning outcomes, and institutional preparedness. Following PRISMA-ScR guidelines, a comprehensive search across ten databases was conducted to identify relevant studies published between January 2020 and December 2025. Forty studies satisfied the inclusion criteria. Pharmacy students in this region responded to the transition to e-learning in diverse ways. While most appreciated the convenience of online modalities, several challenges were consistently enumerated. These were limited technological infrastructure, reduced interpersonal interaction, and disruption of hands-on practical training. Blended learning approaches were largely favored, particularly for their ability to marry online theoretical instruction with face-to-face experiential learning. Reliability and validity issues of internet-based tests were felt by both faculty and students. Stress and mental health problems among students surfaced. Student complaints in general depicted pharmacy education’s need for pedagogic reform, better infrastructure, and student mental health services during e-learning. Areas identified from this review are instructional technology infrastructure improvement, adopting a blended learning strategy, and the need to consider the mental health of students learning at a distance.

## 1. Introduction

The COVID-19 virus pandemic brought about a unique disruption in education globally, impacting more than 1.6 billion learners across 190 countries [1]. Schools had to make a sudden transition from on-ground, face-to-face teaching/learning to hybrid and e-learning modes. Betts et al. [2] referred to this type of transition as “pivotal pedagogy,” contrary to what Hodges et al. termed “emergency remote teaching,” carried out with no preparation and instructional design characteristic of carefully thought-through online learning [2]. The sudden transition posed some problems for health professions education, including pharmacy education that involves intense laboratory, clinical rotation, and patient contact experiential education. Pharmacy education features the integration of science with patient-facing clinical education, and students must achieve proficiency in hands-on skills in compounding, counseling, diagnosis, and public health service. The transition to e-learning immediately raised the issue of how the didactic and practical elements of pharmacy education would be maintained in this crisis [3,4].

Pharmacy education and training programs within the League of Arab States had to find equilibrium in education continuity and competency assurance. The sudden cessation of in-class and on-ground education understandably would affect the practicum training, calling for creative solutions. Student readiness, digital infrastructure, and institutional readiness “to go virtual” would be variable across the region. Prior to the COVID-19 pandemic, pharmacy education across most regions—especially in the Arab League States—relied heavily on conventional face-to-face, on-ground instruction. Didactic courses in pharmacology, medicinal chemistry, and pharmaceutics were complemented by extensive experiential learning, including laboratory work, clinical rotations, and simulation-based case studies. These practical components were central to developing critical thinking, clinical decision-making, and patient care competencies. Furthermore, pedagogical approaches such as team-based learning (TBL) and problem-based learning (PBL) have been long integrated into pharmacy education to promote collaboration and real-world problem solving, thus adding another area of concern in moving to e-learning. Hands-on laboratory training and direct patient interactions during clinical placements were considered irreplaceable aspects of professional formation. Even with attempts to replicate this experience virtually, many students and instructors were skeptical that key practical skills could not be fully developed without in-person, on-ground exposure [5].

Digital tools like Learning Management Systems (LMS) were already present pre-pandemic, but they were largely considered supplemental rather than foundational to pharmacy instruction. As Kahaleh et al. [6] noted, digital platforms were not widely leveraged for clinical skills training, highlighting a gap that would become more pronounced during the pandemic transition [6]. Moreover, global variability in pharmacy education was evident even before COVID-19, shaped by infrastructure quality, regulatory systems, and institutional resources. While high-income countries had access to advanced labs and clinical affiliations, lower-income nations often struggled with inadequate training facilities and limited access to qualified educators. This disparity influenced both the design and delivery of curricula and contributed to differing levels of preparedness for digital transformation [4,7,8].

Some innovation was already underway. Competency-Based Education (CBE) and the integration of emerging technologies like AI-powered drug databases, electronic health records (EHRs), and telepharmacy were beginning to reshape how pharmacy training aligned with evolving healthcare delivery systems [9]. Understanding this pre-pandemic landscape is essential for evaluating the impact of the rapid shift to online and hybrid models. This scoping review synthesizes insights from faculty, students, instructional designers, and administrators to explore how the delivery of pharmacy education evolved during the COVID-19 pandemic, and whether those changes signal temporary adaptation or lasting transformation to e-learning. This review examines the attitudes, motivation, engagement, and adaptability to e-learning among pharmacy students across the League of Arab States, for an all-rounded understanding of the effects of the pandemic and further identification of strategies to enhance the models of future pharmacy education. In particular, this review seeks to:

1. Characterize the attitude, acceptability, engagement, and motivation of pharmacy students toward the use of e-learning during the COVID-19 virus pandemic.
2. Assess the effectiveness of e-learning tools and hybrid models applied to deliver core competencies for students in pharmacy practice.
3. Determine how institutional readiness, digital infrastructure, and educator training enabled this transition.

This scoping review discusses how pharmacy students across the League of Arab States adapted and “dealt with” this sudden shift toward e-learning in the context of satisfaction, motivation, and the efficiency of e-learning models in the wake of the COVID-19 pandemic. This review also investigates the theoretical and practical dimensions in which this transition impacted learning among pharmacy students and highlights both experiential learning challenges and innovations in the region.

## 2. Methods

### 2.1. Eligibility Criteria

Inclusion and exclusion criteria were developed based on the PCC (Population, Concept, Context) framework (see Table 1).

### 2.2. Search Strategy

The scoping review was performed using the PRISMA extension for scoping reviews and the Arksey and O’Malley framework [10,11]. The search was carried out in the winter of 2025 and the results reflect literature published from January 2020 through December 2025. For the context of this review, Arabic-speaking countries are defined as the 22 member countries of the Arab League states [12]. The search was based on ten academic electronic databases (see Table 2). These databases were selected based on the fact that their scopes are related to medical and biomedical aspects. A set of combined terms and phrases were used for the retrieval process (see Table 2). The search strategy was adapted to the indexing system of each database; reference lists of retrieved articles were screened manually for additional studies. All retrieved articles were assessed against the eligibility criteria.

### 2.3. Selection Process

**Figure 1:**
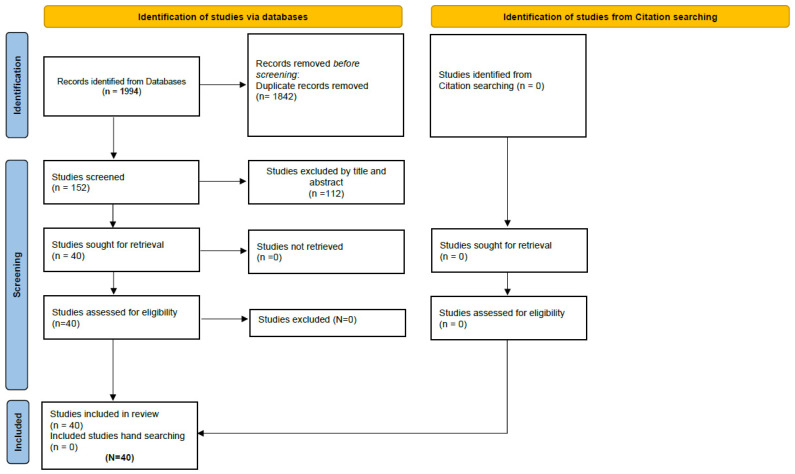
PRISMA Flow diagram.

All retrieved records were imported into Rayyan QCRI software (http://rayyan.qcri.org) to facilitate screening and study selection [13]. Title and abstract screening, followed by full-text review, were conducted independently by two reviewers (BAE and HM) (See Figure 1). Discrepancies were resolved through discussion, with a third reviewer (SG) consulted when consensus could not be reached.

### 2.4. Data Collection and Data Items

Data extraction was performed by one reviewer (HM) using a structured data extraction form. The extracted data included: author(s), year of publication, country, target population, study design, sample size, instructional design, platform used, measurement instruments, outcomes assessed, key findings, and main recommendations. To enhance accuracy, extracted data were reviewed and cross-checked, and any discrepancies were resolved through discussion among the authors.

### 2.5. Data Synthesis

A narrative synthesis approach was used to summarize and describe the characteristics and findings of the included studies. Consistent with scoping review methodology (PRISMA-ScR), findings were synthesized using a structured thematic mapping approach. Extracted data were iteratively grouped into key constructs, and an evidence-mapping process was used to identify patterns across studies. Given heterogeneity in design and outcomes, synthesis was conducted descriptively rather than through quantitative meta-analysis. The resulting thematic constructs were tabulated to enhance transparency of evidence mapping.

### 2.6. Bias Assessment and Limitations

Formal methodological quality assessment was not conducted, as it is not a mandatory component of scoping review methodology [14]. However, several methodological limitations should be noted. The search strategy was developed and executed by a single reviewer and was not formally peer-reviewed, which may introduce a risk of missed studies or confirmation bias. This risk was mitigated through the use of multiple databases, tailored search strategies, manual reference list screening, and independent dual-reviewer study selection.

### 2.7. Ethics Statement

No ethical review oversight was deemed necessary for this review and, therefore, no institutional review board approval was obtained.

## 3. Results

A total of 40 studies were identified and included in this review. Using a structured thematic mapping approach, findings from the included studies were grouped into 10 key constructs to systematically capture cross-study patterns. Across these studies, investigators used questionnaires and focus groups to assess student, faculty, and staff experiences during the rapid transition to e-learning for both didactic instruction and clinical skills training. The research captured the successes, challenges, and limitations of e-learning in pharmacy education across countries with varying levels of technological infrastructure and institutional readiness. Ten key constructs describing the nature and extent of these evaluations, along with associated recommendations, were identified and tabulated (see Supplementary Material Table S1).

### 3.1. University Type and Countries’ SES

These articles, published between 2020 and 2025, provide an overview of how pharmacy education shifted to e-learning during the COVID-19 pandemic. They address student adaptation and satisfaction, instructional design changes, faculty concerns, and the overall effectiveness of online platforms. Of the 40 studies, the majority were conducted in Saudi Arabia (17 studies) and Jordan (10 studies), creating a significant regional imbalance. Countries such as Algeria, Tunisia, Mauritania, Somalia, and Comoros were not represented. The studies included both public and private institutions across different economic contexts.

High-income countries: Institutions in Saudi Arabia [15,16,17,18,19,20,21,22,23,24,25,26,27,28,29,30,31], Bahrain [32], Qatar [33], and the UAE [34,35,36] had well-established digital infrastructures that facilitated smoother transitions to e-learning.

Middle- and low-income countries: Institutions in Lebanon [37], Egypt [38], Jordan [39,40,41,42,43,44,45,46,47,48], Iraq [49,50,51,52], Morocco [53], and Libya [54] faced greater challenges related to internet reliability, faculty preparedness, and student access to digital resources.

Urban vs. rural divide: A clear digital divide was evident, with urban students demonstrating greater technological access and proficiency, while rural students experienced more technical challenges, including limited connectivity and fewer institutional resources.

Taken together, these findings suggest that differences in institutional resources were not merely logistical but shaped the overall quality of the e-learning experience, including student satisfaction, engagement, and perceived learning effectiveness. Studies from high-income settings tended to emphasize optimization and engagement, whereas those from lower-resource contexts highlighted fundamental access and continuity challenges.

### 3.2. Population Studied and Sample Size

The studies included a range of participants, primarily students at different stages of their pharmacy programs, as well as faculty and other university personnel. Sample sizes varied widely, from fewer than 50 participants to more than 2000.

Mid-sized studies: Many studies surveyed between 400 and 1000 participants, providing a balanced perspective on experiences.

Large cross-sectional studies: Some studies, such as Saeed and Almendeel [30], included over 2000 pharmacy students, offering large-scale insights.

Undergraduate vs. postgraduate: Undergraduate students were the primary focus, though some studies examined postgraduate training, including virtual internships (e.g., Almohammed et al. [24]).

Faculty perspectives: Faculty challenges were explored in studies such as Alqurshi [25].

### 3.3. Research Methods and Designs

A range of research designs was used, with cross-sectional surveys being the most common.

Cross-sectional studies (CSS): Representing over 60% of the included research, these studies provided large-scale quantitative data.

Mixed-methods studies (MMS): Combined qualitative and quantitative approaches to provide deeper insights into experiences and adaptations.

Quasi-experimental studies: Some studies (e.g., Ahmed et al. [50]) evaluated hybrid models and learning outcomes.

### 3.4. Instructional Design Approaches

The studies identified several instructional models used in pharmacy e-learning.

Blended learning: Both synchronous and asynchronous approaches were used. Many students preferred hybrid models over fully online formats, particularly for practice-based training [16].

Competency-based instruction: Online learning supported theoretical knowledge but limited hands-on clinical experience [24].

Flipped classroom and project-based learning: These approaches improved engagement and performance in some settings [20].

Simulation-based learning: Virtual simulations were used as alternatives to clinical training, though students reported reduced real-world interaction [43].

Across studies, instructional approaches that incorporated synchronous interaction and hybrid elements were more consistently associated with positive student perceptions, whereas fully asynchronous models were more frequently linked to disengagement and lower perceived learning outcomes.

### 3.5. Faculty Workload and Institutional Adaptations

Faculty workload increased substantially during the transition to online teaching. Studies such as Ali et al. [23] highlighted the need for training in digital tools and assessment strategies. Institutions in high-income settings were better equipped to provide support, whereas those in middle- and low-income countries faced greater challenges.

### 3.6. Student Satisfaction and Choices

Student satisfaction with e-learning varied across studies. While some students valued convenience, access to recorded lectures, and autonomy, others reported dissatisfaction due to reduced interaction and technical challenges. In Saudi Arabia, one study reported that 85% of students preferred to continue e-learning post-pandemic [30]. However, this high level of preference was not consistently observed across other settings. Studies from Jordan and Iraq reported lower satisfaction, particularly among final-year students and those in rural areas with limited internet access [34,39]. Blended learning, combining online theoretical instruction with face-to-face practical components, was the most commonly reported instructional approach [48]. These differences suggest that student preference for e-learning was highly context-dependent, influenced by infrastructure reliability, stage of training, and the extent to which programs incorporated in-person or practice-based components.

### 3.7. Engagement, Motivation, and Interaction

Challenges with motivation and engagement were reported across many studies. Key concerns included reduced peer interaction, home distractions, and limited real-time feedback. One study reported that 39.2% of respondents experienced distractions and 25.3% reported low motivation [17]. Interactive approaches, including synchronous sessions and flipped classrooms (e.g., iFEEL model [20]), improved engagement, whereas asynchronous formats were associated with lower participation. These findings indicate that engagement was less a function of modality alone and more dependent on instructional design features, particularly opportunities for real-time interaction and structured participation.

### 3.8. Trust in Learning and Acquisition of Clinical Skills

Students reported lower confidence in developing clinical skills through e-learning. For example, 43% of students in one study expressed concern about limited patient interaction during virtual internships [24]. Another study reported that over 70% believed online laboratories negatively affected practical skill acquisition [47]. Although telepharmacy and simulation were used as interim solutions, students expressed a preference for in-person training. Despite the use of virtual simulations, the consistent concern across studies suggests that current e-learning modalities are insufficient substitutes for experiential clinical training, reinforcing the importance of hybrid or in-person components in pharmacy education.

### 3.9. Mental Health and Well-Being

All included studies reported increased stress, anxiety, and physical discomfort associated with prolonged screen time and reduced social interaction. Moderate levels of depression and anxiety were observed, particularly among students with limited peer contact and physical activity [41]. Additional studies reported fatigue, musculoskeletal discomfort, and burnout [35]. Female students and those without dedicated study spaces reported higher stress levels. Increased stress, anxiety, and physical discomfort were consistently reported among the studies, largely attributed to prolonged screen time, reduced peer interaction, and disruptions to established learning routines.

### 3.10. Institutional and Faculty Readiness

Institutional readiness varied significantly by country and income level. High-income countries such as Saudi Arabia and the UAE demonstrated stronger digital capacity and faculty support systems. In contrast, in countries such as Iraq, Jordan, and Libya, the transition exposed weaknesses in IT infrastructure, assessment tools, and instructional design. Faculty workload increased due to content digitization and increased student support needs, underscoring the importance of continued investment in technology and training [23].

## 4. Discussion

The pivot to e-learning in response to the COVID-19 pandemic ignited innovation in pharmacy education across Arabic-speaking countries. The dramatic shift required faculty, students, instructional staff, and administrators to find new ways to engage in e-learning—fully or partially—particularly when it came to hands-on clinical training and the development of provider–patient communication and relationship-building skills.

Findings have implications for the infrastructure, teaching/learning process, student engagement strategies, student assessment strategies, and mental health effects of learning at a distance and sometimes asynchronously.

### 4.1. Patient Interaction and Communication Skills

A key gap identified across the reviewed studies is the limited development of patient interaction and communication skills in e-learning environments. While online platforms supported continuity of theoretical instruction, they were less effective in fostering the interpersonal competencies central to pharmacy practice.

Evidence from several studies underscores this concern. For example, students in virtual training settings reported limited confidence in patient-facing skills, with concerns about reduced exposure to direct care [24]. Similarly, the absence of real-time interaction, including non-verbal communication and immediate feedback, diminished the perceived authenticity of training experiences [43] and contributed to a sense of incomplete professional development [33].

These findings suggest that, although simulation and telepharmacy served as useful interim strategies, they do not fully replicate the experiential and relational aspects of in-person training. Communication skills, such as counseling, active listening, and patient engagement, require repeated, high-feedback interactions that are difficult to reproduce in fully virtual formats.

To address this gap, pharmacy programs should more intentionally integrate communication training into blended learning models. Approaches such as standardized patient simulations, structured role-play, and guided reflection within telehealth contexts may help strengthen these competencies. Embedding such activities within both formative and summative assessment frameworks will also reinforce their importance in professional readiness.

Thus, strengthening patient interaction skills should be a priority in post-pandemic curriculum design to ensure that graduates are adequately prepared for real-world practice.

### 4.2. Infrastructure Challenges and Digital Preparedness

Technological infrastructure emerged as a major barrier across many studies. Challenges included limited internet connectivity, inadequate technical support, and restricted access to reliable devices. Several studies reported moderate satisfaction with e-learning, often constrained by technical issues such as network delays and reduced interaction.

Ahmed et al. [50] noted that poor internet connectivity disrupted synchronous components of hybrid learning, while recorded lectures were favored for their accessibility. These findings highlight the need for sustained investment in IT infrastructure and user-friendly learning platforms. Ensuring equitable access to high-speed internet and providing financial support for necessary hardware are critical, particularly in countries with marked socioeconomic disparities such as Jordan, Iraq, and Saudi Arabia.

### 4.3. Blended Learning: The Future-Proof Model

A strong preference for blended learning was observed across the reviewed studies. While fully online education ensured continuity during the pandemic, it often could not support high-quality practical training. For example, Salama and Altaif [48] reported that 75.2% of students preferred a blended model combining online and face-to-face instruction, particularly for laboratory-based courses.

Other studies demonstrated that hybrid models—where theoretical content is delivered online and practical training occurs in person—can effectively balance flexibility and skill development [20,50].

Blended learning addresses many limitations of fully online education while offering adaptability across learning environments. Institutions should therefore develop structured strategies to integrate flipped classrooms, interactive e-lectures, and simulation-based tools into traditional curricula.

### 4.4. Student Motivation and Engagement

Student engagement and motivation were consistently reported as major challenges. Contributing factors included reduced peer interaction, home-based distractions, and limited immediate feedback, all of which affected participation.

For example, Thangam [17] reported that 39.2% of students experienced significant distractions, while others reported low motivation. Additionally, Almhdawi et al. [41] identified moderate levels of depression and anxiety associated with social isolation.

Interactive pedagogical approaches have been used to address these challenges. Shahba et al. [20] found that the iFEEL model significantly improved student engagement and performance, with 75% of students expressing a preference for this approach. These findings suggest that institutions should prioritize faculty training in interactive teaching methods to promote active learning.

### 4.5. Assessment Integrity and Quality Assurance

The transition to e-learning raised concerns regarding the reliability and validity of student assessments, particularly in relation to academic integrity. Studies [25,44] reported instances of academic dishonesty during remote examinations, as well as technical issues that affected assessment quality.

Alternative formats such as open-book examinations introduced additional concerns about accurately assessing student competencies. These findings highlight the need for robust assessment frameworks incorporating proctoring technologies, question randomization, and practical components.

Innovative approaches, such as the use of pre- and post-tests in interactive learning models [20], demonstrate potential for improving assessment validity. Blended assessment strategies may enhance the credibility of online evaluation systems.

### 4.6. Physical and Mental Health Considerations

The shift to online learning was associated with changes in both mental and physical well-being. Studies reported increased stress, anxiety, and physical discomfort related to prolonged screen time and reduced social interaction [35,41].

These effects were compounded by behavioral changes, including reduced physical activity and altered daily routines. Female students and those lacking dedicated study spaces reported higher stress levels.

Institutions should incorporate comprehensive mental health support within academic frameworks. Strategies may include accessible counseling services, promotion of physical activity, ergonomic guidance, and the development of virtual peer-support networks to reduce isolation.

### 4.7. Institutional Preparedness and Leadership

Institutional preparedness played a critical role in the effectiveness of the transition to e-learning. Studies suggest that leadership, organizational culture, and adaptability significantly influenced outcomes [32].

Frameworks such as Ciottone’s disaster cycle [55] help explain how institutional flexibility and resilience enabled more effective responses. Moving forward, leadership should focus on sustaining effective innovations introduced during the pandemic while addressing identified gaps.

Retaining recorded lectures, expanding simulation-based training, and integrating telehealth tools can contribute to the continued advancement of pharmacy education.

## 5. Recommendations

Pre-professional healthcare education should adopt long-term, student-centered strategies that integrate experiential learning with advanced technology. The following recommendations address key areas for improvement.

### 5.1. Strengthening Infrastructure and Technology

Institutions should invest in digital infrastructure to ensure equitable access and effective delivery of online education. Key priorities include:

- Expanding access to high-speed internet, particularly in underserved areas;
- Enhancing e-learning platforms with interactive case studies and simulations;
- Strengthening cybersecurity measures to protect data integrity.

Future research: Explore the role of AI-driven tools in improving engagement and learning outcomes.

### 5.2. Supporting Digital Learning for Faculty and Students

Effective online education depends on the digital readiness of both faculty and students. Institutions should:

- Provide faculty development programs in e-learning methodologies and digital tools;
- Offer student orientation on navigating online platforms and maintaining academic integrity.

Future research: Examine the impact of ongoing faculty development on learning outcomes.

### 5.3. Advancing Blended Learning

Blended learning should be a central model in pharmacy education. Institutions should:

- Implement flipped classroom approaches;
- Use problem-based and team-based learning strategies;
- Expand virtual labs and clinical simulations.

Future research: Assess long-term impacts of blended learning on competency development.

### 5.4. Enhancing Assessment Strategies

To improve assessment quality in virtual settings, institutions should:

- Develop competency-based assessment tools;
- Use learning analytics to monitor student performance;
- Incorporate frequent formative assessments;
- Improve remote proctoring systems.

Future research: Evaluate adaptive assessment technologies.

### 5.5. Supporting Mental Health and Well-Being

Institutions should prioritize mental health by:

- Establishing virtual peer-support systems;
- Providing accessible counseling services;
- Promoting balanced workloads;
- Educating faculty to recognize and respond to student distress.

Future research: Investigate long-term effects of mental health interventions on academic outcomes.

This study contributes to the literature in several distinct ways. First, it examines a unique and underreported event within the context of the Arab League, a setting that remains comparatively understudied in pre-licensure pharmacy education. Second, rather than pursuing cross-professional or cross-national comparisons, this analysis prioritizes depth over breadth, offering a detailed and grounded account of processes that are often treated abstractly in broader reviews. Third, the study draws on a panoramic analysis of evaluation characteristics across programs, providing insight into pharmacy education practices that are not yet well captured in the existing literature.

## 6. Limitations

Limitations in these studies were evident. The methodologies were not uniform, and hence any comparison was not as robust. Most of the research depended on self-report data, which is vulnerable to bias, overrepresenting the high-achieving students and omitting accounts of disengaged or marginalized learners. Most of the research was focused on the short-term impacts that online education had during the very beginning of the pandemic, with very limited exploration of longer-term outcomes such as practical skills development or career readiness. Equally underrepresented were the views of staff, another important constituent in any holistic understanding of challenges related to online education. Geographically, many such studies have a certain region of focus, and thus generalizing such findings across other parts of the world with different educational systems and technological infrastructures may be inappropriate. One included study was conducted in a multi-national setting outside the Arab region but was retained due to its relevance to pharmacy education during the COVID-19 e-learning transition; however, its findings may not fully reflect region-specific experiences. Very little attention has been paid to access to digital tools and financial barriers, which constitute some of the most critical factors affecting learning experiences.

## 7. Conclusions

The COVID-19 pandemic necessitated a rapid transition to e-learning in pharmacy education, prompting widespread curricular and instructional adaptation across the included countries in this review. While the evidence base is geographically concentrated and does not fully represent all League of Arab States member countries, consistent patterns emerged across diverse settings regarding both the benefits and challenges of this transition.

Institutions must embrace integrative and flexible solutions that leverage the advantages of online learning while addressing its limitations. Staff development, mentoring, and sustained investment in digital infrastructure are essential to support teaching effectiveness, student well-being, and long-term sustainability. Blended learning emerged as a key model, combining the strengths of online theory with in-person experiential training.

Although these findings are drawn from a subset of countries, they provide transferable insights that may inform pharmacy education in similar contexts, particularly in settings undergoing digital transformation. At the same time, the absence of studies from several Arab countries highlights a critical gap in the literature and underscores the need for future research in underrepresented regions to ensure more equitable and comprehensive regional understanding.

Synthesis of findings from these studies yields the following recommendations for pharmacy education development:

➢ Infrastructure: Improve IT systems and ensure equitable access to digital tools.
➢ Blended Models: Integrate online theoretical instruction with hands-on practical learning.
➢ Engaging Learning: Promote interactive pedagogies such as flipped classrooms.
➢ Assessment: Develop robust and transparent evaluation methods, including practical competencies where feasible.
➢ Mental Health: Expand student support services addressing psychological and physical well-being.
➢ Institutional Planning: Strengthen leadership and preparedness for future disruptions.

## Figures and Tables

**Table 1:** PCC (Population, Concept, Context) Criteria for inclusion and exclusion of studies.

Parameter	Inclusion Criteria	Exclusion Criteria
Population	University pharmacy students that reside in an Arab League member state *	N/A
Concept/ Context	Pharmacy students’ opinion, satisfaction, perception, challenges, acceptance, hesitancy, or attitude on e-learning during COVID-19 pandemic.	N/A
Language	English, Arabic, or French	All other languages
Study Type	Peer-reviewed original research articles (quantitative or qualitative)	Non-Peer-reviewed articles Study protocols Narratives Similar article types Gray literature Communications White papers Theses/Dissertations

* “Algeria”; “Egypt”; “Bahrain”; “Comoros”; “Djibouti”; “Iraq”; “Jordan”; “Saudi Arabia”; “Kuwait”; “Lebanon”; “Libya”; “Mauritania”; “Morocco”; “Oman”; “Palestinian Territories”; “Qatar”; “Yemen”; “Somalia”; “Sudan”; “Syria”; “Tunisia”; “the United Arab Emirates”. N/A—Not applicable.

**Table 2:** Electronic Databases Used with Relevant Search Period and Terms.

Databases	Search Period	Keywords, Search Terms, and Phrases
Scopus, PubMed, SpringerLink, Wiley Online, ProQuest, ArticleFirst, EBSCOhost, Taylor & Francis, Web of Science, and ScienceDirect	2020 through 31 December 2025	“novel coronavirus” [All Fields]; OR “coronavirus 2019” [All Fields]; OR “COVID 2019” [All Fields]; OR “COVID19” [All Fields]; OR “COVID-19” [All Fields] OR “SARS-CoV-2” [All Fields] OR “HCoV-19” [All Fields] OR “2019-nCoV” [All Fields] AND “opinion” OR “satisfaction” OR “perception” OR “attitude” [All Fields] AND “university students” [All Fields]; OR “college students” [All Fields]; OR “higher education students” [All Fields]; OR “post-secondary education students” [All Fields] AND “Pharmacy” OR “Pharmacist” [All Fields] AND “distance learning” or “remote learning” OR “e-learning” OR “online learning” OR “online education” OR “online training” OR “virtual learning “ OR “distant learning” [All Fields] AND “Algeria”; “Egypt”; “Bahrain”; “Comoros”; “Djibouti”; “Iraq”; “Jordan”; “Saudi Arabia”; “Kuwait”; “Lebanon”; “Libya”; “Mauritania”; “Morocco”; “Oman”; “Palestinian Territories”; “Qatar”; “Yemen”; “Somalia”; “Sudan”; “Syria”; “Tunisia”; “the United Arab Emirates” [All Fields]

## Data Availability

All data generated and analyzed during this review are included in the published review article.

## Supplementary Materials

The following supporting information can be downloaded at: https://www.mdpi.com/article/10.3390/pharmacy14040099/s1, Table S1: Summary of study findings (n = 40) [15,16,17,18,19,20,21,22,23,24,25,26,27,28,29,30,31,32,33,34,35,36,37,38,39,40,41,42,43,44,45,46,47,48,49,50,51,52,53,54].
