# Supplementary material for: Pharmacy Students’ Perception of E-Learning During the COVID-19 Pandemic Across the League of Arab States: A Regional Scoping Review
Source: Pharmacy (Basel). 2026 Jul 3;14(4):99. doi: 10.3390/pharmacy14040099 (PMC13414875; doi:10.3390/pharmacy14040099)
Supplement: Supplementary file 1 [file pharmacy-14-00099-s001.zip › pharmacy-4348770-supplementary.pdf]

Table S1. Summary of study findings (n = 40).

Authors/ Year	Country	Target Population/Sample Size	Type of Study	Instructional Design	Platform Used	Measurement Instruments	Measured Parameters	Main Results/Findings	Main Recommendations
**Abdel Rahman et al. (2022) [34]**	UAE	Undergraduate Pharmacy (1655 students with 1353 valid responses)	Descriptive field study using a quantitative approach.	Descriptive evaluation of online learning implementation, using a structured questionnaire with 40 items divided into different aspects (readiness, challenges, and improvements).	Various Online Teaching & Online Platform	A validated and reliable questionnaire based on a 5-point Likert scale, reviewed by academic experts and tested for reliability (Cronbach's alpha: 0.886).	1. Readiness, training, and technical support for online learning. 2. Satisfaction with teaching and learning processes. 3. Challenges faced in online learning. 4. Suggestions for improving online learning.	Readiness and Support: Rated high by students (mean 3.73). Satisfaction: Moderate satisfaction with online teaching (mean 2.82). Challenges: Weak technical skills of both students and faculty, technical issues like network delays, and reduced direct interaction rated moderate (mean 3.25). Improvement Suggestions: Strong demand for continuous technical support	1. Increase technical and pedagogical training for faculty members. 2. Improve the IT infrastructure for seamless online learning. 3. Offer technical and financial support for students lacking resources. 4. Develop a comprehensive strategy for blended or fully online education as a contingency for future disruptions.

							and regular updates to learning platforms (mean 3.62).	
**Alghamdi and Ali (2021) [15]**	Saudi Arabia	Pharmacy (241 students)	CSS	Online learning using revised teaching and assessment plans by faculty and preceptors to maintain learning outcomes and academic integrity during the COVID-19 lockdown.	Rafid (a locally developed Learning Management System)	Self-administered bilingual questionnaire (English and Arabic) with five key domains assessed using 5-point Likert scales.	Technology access, online skills, motivation, preferences between online and face-to-face learning, and online versus face-to-face examinations.	Students reported high access to technology and strong online skills.<br>Male students showed a significantly more favorable attitude towards online exams compared to females.<br>Internship students had the lowest overall satisfaction scores, while first-year students had the highest.<br>Many students preferred online exams for reduced stress but felt they	1. Improve support and design for experiential learning, particularly for internship-level students.<br>2. Provide training for students on time management in online learning environments.<br>3. Conduct further studies on the actual impact of online education on student learning outcomes beyond self-reported perceptions.

							learned less through online education. Over half of the students cited the need for more training in time management for online learning.	
**Abu-Huwaij et al. (2022) [39]**	Jordan	Undergraduate Pharmacy (573 students out of a population of 1500)	CSS	Delivered using Moodle for course materials and Microsoft Teams for interactive sessions. Included both synchronous and asynchronous elements. Evaluation of five quality aspects: course content delivery, online interaction, technology access, assessment, and challenges.	Various Online Teaching & Online Platform	Self-administered questionnaire based on a 5-point Likert scale. Internal reliability measured with Cronbach's alpha (0.85).	1. Satisfaction with course content delivery 2. Online interaction quality 3. Technology access and ease of use 4. Assessment credibility and effectiveness 5. Challenges encountered during distance learning	Overall satisfaction was acceptable (Mean: 3.33, 66.6%). Course content delivery was effective but practical skills were harder to meet. Fifth-year students were more satisfied compared to junior students. Mobile devices (especially iPhones) were widely used, with challenges for Android users.	1. Provide internet and device support for students in need. 2. Address challenges faced by junior students through mentorship and technical training. 3. Enhance assessment credibility and fairness. 4. Develop an online "Student Affairs Office" for counseling and problem-solving.

								Challenges included internet quality, strain symptoms, and lack of interaction. Assessment was viewed as credible but raised concerns about academic integrity.	5. Focus on proactive strategies to support sustainable online education.
**Ahmed et al. (2020) [49]**	Iraq	Undergraduate Pharmacy (1128 undergraduate, 113 postgraduate students)	Position report on the transition to distance learning	Transition from face-to-face to full virtual education using Google Classroom and other platforms. Synchronous and asynchronous modes of teaching with video lectures, PowerPoint slides, and live discussions via Google Meet, Zoom, and Free Conference Call.	Online Platform	Feedback from students via surveys conducted after the first semester of virtual learning.	1. Students' perceptions of e-learning effectiveness.<br>2. Participation in virtual classrooms and exams.<br>3. Technical and logistical challenges during the transition.	60% of students considered e-learning a good solution during the pandemic but did not prefer it as an adjuvant to traditional learning post-pandemic. Student participation in virtual classrooms reached 100% by the second semester after	1. Continue integrating e-learning components such as hybrid models and flipped classrooms.<br>2. Strengthen IT infrastructure and support for students and faculty.<br>3. Implement strategies to improve student acceptance and engagement with

				Adaptations included simulated patient-pharmacist consultations and video demonstrations for practical components.				improved organization and technical support. Significant challenges included internet connectivity issues and the sudden shift to online exams. A majority of students preferred better internet services to enhance e-learning experiences.	online education. 4. Provide technical training for faculty and students to ensure effective use of online platforms. 5. Use the lessons learned to optimize future educational strategies for emergencies.
**Ahmed et al. (2021) [50]**	Iraq	Pharmacy students (548 students out of 1370)	Quasi-experimental, post-intervention evaluation of the hybrid education model	Hybrid education model with virtual lectures and in-class exams. Labs delivered in a hybrid mode: lecture components online, practical components on-campus.	Various Online Teaching & Online Platform	End-of-semester evaluation survey using a 5-point Likert scale. Measures included student satisfaction with hybrid education,	1. Satisfaction with synchronous and asynchronous lectures. 2. Perceptions of lab and practical session effectiveness. 3. Comparisons of hybrid education vs. fully online education.	Most students agreed that hybrid education was better than fully online education for lab-based courses. In-class exams were perceived to improve scientific gain more effectively than	1. Maintain and enhance the use of Google Classroom and university email for communication and content delivery. 2. Integrate flipped classroom models and

			Synchronous and asynchronous lecture delivery, with recorded lectures made available on YouTube for flexibility.		virtual delivery, online exams, and support services.	4. Use of Google Classroom and university email for communication. 5. Satisfaction with online exam management and technical support. 6. Perception of faculty understanding during the pandemic.	online exams. Skills in using Google Classroom and taking online exams improved significantly compared to the previous academic year. Poor internet services hindered the effectiveness of synchronous lectures, with students preferring recorded video lectures. Median responses indicated neutral satisfaction with overall hybrid education implementation.	hybrid components in traditional courses. 3. Address resource limitations such as poor internet infrastructure and lack of exam monitoring tools. 4. Provide ongoing training for faculty and students on ICT tools to improve education quality. 5. Leverage virtual platforms for international collaborations and knowledge sharing.
**Al Zahrani et al. (2021) [16]**	Saudi Arabia	Health Science (1288 students)	CSS	Conducted fully online education due to the COVID-19	Various Online Teaching &	Validated questionnaire (22 closed-ended	1. Technical preparedness for online learning. 2. Students'	58.2% of students agreed they had sufficient information about	1. Adopt blended learning strategies combining online

		pandemic. Platforms used included Blackboard Collaborate and Zoom for synchronous and asynchronous learning. Questionnaires included sections on technical preparedness, academic achievements, interaction, feedback, and assessment methods.	Online Platform	questions on a 5-point Likert scale, 7 open-ended questions). Reliability tested with Cronbach's alpha value of 0.93.	satisfaction with course content delivery and accessibility.<br>3. Interaction and feedback with instructors and peers.<br>4. Perception of online practical sessions and overall course quality.<br>5. Evaluation of the assessment methods during online learning.	online platforms, but only 42% felt technical support was adequate. Only 30% agreed the quality of online teaching was comparable to traditional classes, with 56.1% finding online learning unsuitable for health sciences. Practical sessions were deemed ineffective by 43.7%, emphasizing the inadequacy of virtual labs for hands-on training. Interaction with instructors and peers during e-learning was adequate for 49.8% and 57.3%	lectures with in-person practical/clinical training.<br>2. Provide extensive training for educators and students in e-learning technologies.<br>3. Address infrastructure challenges like poor internet connectivity and inadequate hardware.<br>4. Establish clear and unified policies for online course delivery and assessment.<br>5. Incorporate longer and more flexible exam schedules with anti-cheating mechanisms.<br>6. Foster active

							of students, respectively. Assessment clarity was agreed upon by 52.1%, but fairness was questioned by 42.6%. Stress associated with e-learning was noted by 41.8%, while overall satisfaction was moderate (41.5%).	interaction between students and instructors to enhance engagement.
**Thangam (2023) [17]**	Saudi Arabia	Undergraduate Healthcare (237 students)	CSS	Online learning experience during the COVID-19 pandemic, focusing on satisfaction levels and challenges encountered	Online Platform	Structured questionnaire with a satisfaction scale (5 subsections) and challenges scale (10 items)	Levels of satisfaction with online learning across lecturer interaction, course delivery, learning environment, and assessment. Challenges including technical skills, distraction, stress, practical learning,	Overall satisfaction was moderate (Mean = 55.97, SD = 10.97, 75%). Students rated learning environment and lecturer interaction highest but reported lower peer support satisfaction.	Enhance the design and infrastructure of online education to better support clinical skills learning. Address common challenges through student training, improved peer support systems,

	and interpreting clinical concepts.	Challenges were low overall (Mean = 7.50, SD = 6.69, 19%). Common challenges included distraction (39.2%), stress (30.8%), lack of motivation (25.3%), and difficulty interpreting clinical concepts (17%). Female students reported higher satisfaction levels, and students using laptops were more satisfied than those using mobile phones. A negative correlation was observed between satisfaction and	and reliable IT infrastructure. Develop blended learning models to integrate the strengths of online and traditional learning methods. Prepare students and faculty for future transitions to online learning with tailored orientation programs.

							challenges ($p < 0.001$).	
**Geddawy et al. (2023) [18]**	Saudi Arabia	Undergraduate Health Sciences (330 students)	CSS	A structured questionnaire assessing knowledge, attitudes, perceptions, and learning satisfaction related to COVID-19.	Online Platform	Questionnaire constructed from previous studies, validated with Cronbach's alpha values (0.879 for English, 0.83 for Arabic versions).	Knowledge of COVID-19, attitudes toward preventive measures, learning satisfaction, and willingness to receive the COVID-19 vaccine.	High knowledge levels about COVID-19 (mean score: 5.63/6). Positive attitudes toward COVID-19 prevention but moderate learning satisfaction (mean score: 11.28/19). Younger students reported higher learning satisfaction. Students with chronic diseases had lower attitude scores and vaccine willingness.	Enhance online learning platforms, improve clarity in study plans, provide better support for students with chronic diseases, and incorporate feedback to address learning satisfaction and stress.
**Cassidy et al. (2024) [32]**	Bahrain	Senior leaders, staff, and students from a Health Sciences (105 = 4 senior	Qualitative case study	Analysis of transition to online learning and hybrid models during the pandemic	Various Online Teaching	Semi-structured interviews and focus groups with thematic	Institutional preparedness, response, recovery, and mitigation/prevention during the	IT infrastructure, organizational culture, and facilitative leadership were critical for	Institutions should plan proactively for crises using frameworks like Ciottone's

		leaders (interviewed), 53 staff, and 48 students (focus groups)		through the lens of Ciottone’s disaster cycle		framework analysis	pandemic; factors affecting operational changes and leadership roles	adapting to the pandemic. Students faced challenges like loss of social interaction and clinical exposure but appreciated innovative teaching methods. Staff demonstrated adaptability and creativity but experienced challenges with rapid skill acquisition and balancing safety with education.	disaster cycle. Leadership should emphasize core values to foster purpose among students and staff. Retain beneficial innovations from the pandemic and avoid reverting to outdated methods post-crisis. Develop flexible and resilient digital infrastructure to ensure continuity in education during future disruptions.
**Al-Mansouri et al. (2024) [51]**	Iraq	Undergraduate (882 Students)	CSS	Online education delivered via platforms such as Google Classroom and Zoom. Focused on asynchronous learning methods	Various Online Teaching	A standardized online questionnaire with closed-ended (Likert scale) and open-ended	1. Technical preparedness and challenges in online education. 2. Student satisfaction with online learning. 3. Barriers faced	Technical challenges: 77% faced difficulties with internet access or poor-quality services; 91.6% found existing platforms	1. Improve internet infrastructure and ensure student access to reliable devices. 2. Train faculty in online teaching

		with opportunities for discussion and participation.		questions to capture perceptions of online learning challenges and barriers. Data analyzed using descriptive statistics.	by students and faculty. 4. Perceptions of online platforms' effectiveness. 5. Recommendations for future integration of online education.	inadequate for student needs. Student-related barriers: Limited interaction with faculty and peers, lack of technical expertise, and prolonged study hours reported by a significant proportion. Faculty-related challenges: 71.4% highlighted inadequate digital skills among educators; 68.2% noted low interest in online teaching, with disorganized timetables cited by 65.6%. Content delivery: 83.6% of students reported that clinical and laboratory	methodologies and tools. 3. Address disorganized timetables and provide technical support for educators and students. 4. Incorporate blended learning strategies to combine in-person practical training with online content. 5. Develop user-friendly and effective online platforms tailored to the needs of medical education.

								courses could not be effectively conducted online. Only 18.9% were satisfied with online learning; however, 49.4% recommended continuing online education blended with classical methods post-pandemic.	
**Shahba et al. (2022) [20]**	Saudi Arabia	Pharmacy (158 students)	MMS	Blackboard, Storyline 360, Camtasia, Google Forms.	Various Online Teaching & Online Platform	Online Tests: Pre-tests and post-tests were conducted via Blackboard® to evaluate students' comprehension before and after the lectures. Questionnaires: Surveys were distributed to	Students' Scores: Pre-test, post-test, and comprehensive exam scores were used to measure learning outcomes and comprehension. Attendance and Participation: Average attendance rates were recorded for both PICkLE and iFEEL activities.	iFEEL (interactive flipped e-learning) improved mean post-test scores (95.2%) compared to remote PICkLE (93.5%) and live PICkLE (90.2%). 92% of students and 85% of faculty provided positive feedback on e-lectures. 75% of students preferred iFEEL	1. Adopt iFEEL as a part of hybrid learning strategies for improved student engagement. 2. Invest in faculty training and tools to streamline e-lecture preparation. 3. Evaluate long-term impacts of iFEEL on knowledge

						students and faculty members to gather their perceptions on the quality and effectiveness of learning models. Open-book Exams: Conducted individually or in groups as part of the classroom activity.	Qualitative Feedback: Students and faculty evaluated aspects such as clarity of material, usefulness of audio and visual tools, and overall satisfaction with the models. Preference and Engagement: Metrics such as preference for teaching models and motivation levels were assessed using Likert scale-based survey responses.	over PICkLE for future courses, highlighting better visualizations and engagement. Challenges included technical issues and time-consuming preparation for faculty.	retention and performance. 4. Expand interactive e-lectures to other courses requiring visual and practical components.
**Al-Neklawy and Ismail (2022) [22]**	Saudi Arabia	Sciences students (MBBS Program: 157 year two and 149 year three students Nursing	Descriptive evaluation of online team-based learning	Pre-class preparation with materials (slides, readings, videos) provided on Blackboard. Online TBL sessions included	Various Online Teaching	Post-session survey with 30 Likert-scale questions (1 to 5 points) assessing recall, engagement,	1. Students' preparation and readiness. 2. Engagement and collaboration within teams. 3. Satisfaction with TBL as a learning	All programs showed significant positive responses compared to neutral responses. No significant	1. Implement online TBL for other medical curricula to enhance active learning. 2. Improve blended learning

		Program: 53 students Doctor of Pharmacy (PharmD): 25 students Medical Laboratory Sciences (MLS): 11 students)	(TBL) sessions	individual readiness assurance tests (IRAT) and team readiness assurance tests (TRAT) via breakout rooms. Application exercises and team discussions followed by instructor-led clarification. An orientation video prepared for students and instructors.		and satisfaction.	method. 4. Effectiveness of Blackboard Collaborate in facilitating TBL.	differences in mean responses across different programs (*p* > 0.05). Students found online TBL effective for understanding course material and engaging in discussions.	by integrating virtual TBL into regular teaching practices. 3. Conduct further studies to evaluate long-term academic outcomes of online TBL. 4. Ensure adequate preparation and orientation for both students and faculty.
**Al-Alami et al. (2022) [40]**	Jordan	Pharmacy (442 students surveyed, with 402 included)	CSS	Theoretical anatomy and histology courses delivered entirely online during the COVID-19 lockdown. Content included lectures provided through platforms like Microsoft	Various Online Teaching & Online Platform	A validated questionnaire with 45 items, distributed via Google Forms, covering demographics, perceptions of the educational	1. Students' perceptions of the educational process. 2. Strengths of remote learning. 3. Challenges faced during remote learning. 4. Impact of	Strengths: Flexibility in time and location, ability to review recorded lectures, and enhanced collaboration with peers. Challenges: Lack of face-to-face interaction,	1. Develop blended learning approaches to balance theoretical and practical learning needs. 2. Record livestream lectures for student review

				Teams and Moodle, with livestream and recorded lecture options. No practical components (e.g., physical models or cadaveric dissections) included.		process, strengths, challenges, and study habits. Likert-scale questions with reliability confirmed by Cronbach's alpha (0.858).	remote delivery on study habits.	inadequate internet access, and limited availability of practical learning resources. Median perception score was 3.57, with significant challenges related to technical issues and internet reliability. 40.8% of students felt the online course contributed positively to understanding course content, while others emphasized the theoretical nature of the course as a limitation.	and provide detailed tutorials for online platforms. 3. Improve internet infrastructure and access to educational resources. 4. Encourage educators to create interactive content and adapt teaching methods to online platforms. 5. Evaluate the long-term impact of remote learning on academic outcomes and course retention.
**Ali et al. (2021) [23]**	Saudi Arabia	Pharmacy (790 students)	Qualitative case study	Courses shifted fully online during the COVID-19	Various Online Teaching &	Thematic analysis of student responses	1. Facilitators for online education during lockdown 2. Barriers to	Facilitators: Time saved due to no commuting, flexibility in	1. Incorporate hybrid learning models combining online

		pandemic lockdown. Platforms included Blackboard, Zoom, and others, with a focus on synchronous and asynchronous learning. Assessment methods included online exams and alternative assignments.	Online Platform	collected during a three-day structured Twitter chat. 29 validated questions distributed into four main topics: learning and assessment, online exams, use of technology, and lessons learned.	online education during lockdown 3. Comparison of online vs. onsite education 4. Role of technology in online learning 5. Suggestions for improving online education 6. Long-term impacts of online education	attending lectures, availability of recorded sessions, and improved student technology skills. Barriers: Poor internet connectivity, inadequate interaction, unsuitable scheduling of live sessions, increased assessments, and challenges with online practical work. Online vs. Onsite: Onsite education was preferred for practical and hands-on training, but online education was viewed as convenient and	lectures with onsite practical training. 2. Provide technical training for educators to enhance digital skills. 3. Address internet connectivity issues and ensure fair distribution of assessments. 4. Record live lectures for student review while balancing live participation. 5. Develop innovative assessment strategies to discourage cheating and promote deeper learning.

							less stressful. Technology: Mixed responses on the effectiveness of platforms like Blackboard and Zoom; some faculty needed better technical training. Long-term impact: Improved GPAs, development of time management and self-learning skills, but reduced opportunities for practical and oral communication skill development.	
**Almhdawi et al. (2021) [41]**	Jordan	Undergraduate Science (485 students)	CSS	Distance learning implemented entirely online during the COVID-19	E-learning & Online Platform	Self-administered online questionnaire Health-related	1. HRQoL scores (physical and mental components) 2. Levels of	Physical HRQoL mean score: 66.5, Mental HRQoL mean score: 44.8 Moderate levels	1. Address physical and mental health challenges of students during

	pandemic lockdown. Courses delivered using platforms for synchronous and asynchronous learning.	Quality of Life (HRQoL) measured using the SF-12 surveyMental health status assessed with the Depression Anxiety Stress Scale (DASS-21) Physical activity evaluated with the International Physical Activity Questionnaire (IPAQ) Musculoskeletal health assessed with the Neck Disability Index (NDI)	depression, anxiety, and stress 3. Physical activity and musculoskeletal health 4. Satisfaction with online learning	of depression and anxiety, mild levels of stress reported by students Satisfaction with online teaching was low (mean score: 2.3 on a 5-point Likert scale). Factors negatively associated with HRQoL: depression, stress, and neck pain. Factors positively associated with HRQoL: physical activity, satisfaction with online learning, and weekly study hours.	emergencies. 2. Improve ergonomics in online learning settings to reduce musculoskeletal issues. 3. Enhance the quality and satisfaction of online teaching. 4. Encourage physical activity and healthy study habits among students. 5. Integrate mental health support into academic planning during future disruptions.

**Almohammed et al. (2021) [24]**	Saudi Arabia	Undergraduate pharmacy (130 eligible students for IPPE (87 responded to the questionnaire)CSS 7 APPE students participated in training design and implementation)	Virtual IPPE program replacing traditional in-hospital training. Delivered using the university's LMS with asynchronous pre-recorded lectures, simulation-based activities, and online courses. Designed to achieve most training objectives, excluding those requiring direct patient interaction.	Various Online Teaching & Online Platform	IPPE Students' Experience Questionnaire (48 items, including demographics and 40 Likert-scale questions on virtual training experience). Open-ended qualitative questionnaire for APPE students.	1. Student satisfaction with virtual IPPE.<br>2. Perceived benefits and challenges.<br>3. Effectiveness of simulation activities and assessments.<br>4. Perceived shortcomings of virtual training.	60% of IPPE students were satisfied with their overall experience, citing flexibility and simulation activities as positive aspects. 43% expressed concerns about inadequate direct patient care experience. 70% believed the virtual training helped them apply previously learned concepts. Main challenges included finding reliable internet and difficulty with team-based activities. APPE students valued their role in designing and administering the	1. Incorporate a mix of virtual and traditional training to address gaps in direct patient care.<br>2. Utilize telemedicine and advanced simulations to improve clinical skills.<br>3. Continue engaging APPE students in training design for peer-based learning.<br>4. Ensure robust IT infrastructure and reliable internet access for all students.<br>5. Conduct further studies to evaluate long-term effectiveness

							program, gaining academic and teamwork skills.	of virtual training programs.
**Alqurshi (2020) [25]**	Saudi Arabia	Pharmacy students and faculty members (703 pharmacy students from 19 different colleges 74 faculty members from 10 different colleges)	CSS	Remote teaching implemented using virtual classrooms via platforms like Blackboard and Microsoft Teams. Assessment shifted to alternative methods such as online exams, essays, and oral presentations.	Various Online Teaching & Online Platform	Two questionnaires (student- and teacher-focused), each using a 5-point Likert scale to assess satisfaction with virtual classrooms, course learning outcomes (CLOs), and alternative assessments. Individual interviews with three students and four teachers to clarify survey responses.	1. Effectiveness of virtual classrooms in delivering course content.<br>2. Completion of CLOs during the lockdown.<br>3. Appropriateness and challenges of alternative assessment methods.<br>4. Student satisfaction with remote learning.	Over 60% of teachers found it challenging to deliver complex scientific concepts remotely. Limited student-teacher and student-student interactions were reported as a major issue by both groups. Approximately 20% of teachers and 35% of students believed CLOs were not fully achieved during the lockdown. Alternative assessments (e.g., essays and oral exams) were considered	1. Addressing CLO Gaps: Conduct revision sessions or short courses in the following semester to cover missed learning outcomes.<br>2. Contingency Planning: Develop a contingency course specification (CCS) outlining strategies for remote teaching and assessment during emergencies.<br>3. Improving Assessments: Train students and faculty on alternative

			beneficial by 70% of teachers but challenging for students due to lack of experience and unclear instructions. Student marks during the lockdown were significantly higher, raising concerns about the accuracy of assessments in reflecting CLOs.	assessment methods and ensure clear marking criteria. 4. Enhancing Interaction: Utilize discussion forums and collaborative tools to improve student-teacher and peer interactions. 5. Infrastructure Improvements: Strengthen internet and technology support for effective remote learning. 6. Future Strategies: Incorporate hybrid learning models and integrate LMS tools into daily

									teaching practices.
**Alsahali et al. (2024) [26]**	Saudi Arabia	Pharmacy (141 students)	CSS	Distance learning platforms with synchronous and asynchronous modes, including Blackboard and Zoom.	Various Online Teaching & Online Platform	Web-based survey consisting of 34 questions across six sections.	Students' attitudes, preferences, effectiveness of distance learning, communication modes, social engagement, and emotional responses.	Majority did not wish to continue distance education for laboratory courses and team-based learning after the pandemic. Distance courses were found simple by 83.7%, but 58.9% reported impaired social engagement on campus. Loss of face-to-face interaction was the most significant drawback.	Educational institutions should balance the advantages of distance learning with the need for peer and instructor interaction, considering student preferences for effective educational strategies.
**Altwaijry et al. (2021) [27]**	Saudi Arabia	PharmD students & academic staff (223 students and 38	MMS	Full distance learning during lockdown and blended learning afterward.	Various Online Teaching & Online Platform	Surveys with Likert-scale responses and focus group discussions	Readiness for distance education, perception, barriers, and	Positive views on readiness for the shift to distance learning. Perception	Enhance communication and interaction in online education. Improve training

		academic staff)					acquisitions due to distance learning	toward distance education was mostly positive but included challenges like reduced quality of interaction. Key barriers included technical issues, reduced communication quality, and prolonged screen time. Acquisitions included enhanced independent learning and teaching skills.	on time management and technology use. Address technical and personal barriers to optimize distance learning.
**Alwassil et al. (2024) [28]**	Saudi Arabia	First-year (P1) pharmacy (196 students)	Causal-comparative study	Online classes integrated virtual platforms, PowerPoint presentations, case-based discussions, and open-book activities.	Various Online Teaching	Midterm and final exams, case discussions, and satisfaction surveys	Learning performance (exam scores), engagement, preparedness, ability to answer questions, professionalism,	The mean score for online students was 80.45 ± 12.65, higher than 71.90 ± 9.50 for on-site students (p = 0.0001). Online tools positively	Enhance virtual learning tools and technical support. Address student engagement and motivation challenges in online settings. Conduct further

						and psychomotor skills	impacted students’ scores, with improved flexibility and participation. Students expressed challenges like home distractions, isolation, and technical issues.	studies to refine blended learning strategies for medicinal chemistry education.
**Barakat et al. (2022) [42]**	Jordan	Medical, pharmacy, nursing, dentistry, and CSS veterinary medicine (939 students)	Fully online education using distance learning platforms during the COVID-19 pandemic	Online Platform	Online survey including sociodemographic, perception, and barrier assessment questions	Student perception, satisfaction, barriers to online learning, and use of e-learning tools.	54.3% of students reported unsatisfactory/very unsatisfactory experiences with online learning. Weak internet connection (80.4%) and lack of motivation (77.0%) were key obstacles. Students preferred face-to-face communication (72.6%). Private	Address technical barriers like internet access and reliability. Implement new pedagogies to improve engagement and student motivation. Provide training for both students and instructors on online tools.

								university students and males showed more positive perceptions. Median perception score: 2.4/5.	
**Barakat et al. (2023) [43]**	Jordan	Senior pharmacy (109 students)	CSS	Virtual community pharmacy training using pre-recorded videos by certified preceptors covering key pharmacy topics	Online Platform	Validated online questionnaire including benefits, barriers, and suggestions for improving virtual training.	Student perceptions, benefits, barriers to virtual training, and suggestions for improvement.	54.1% reported increased involvement with the pharmacy profession. 46.8% found it beneficial for time management, and 56.0% reported improved IT skills. The most significant barrier was the absence of eye contact (52.3%). 51.4% agreed that combining virtual and conventional methods would enhance training outcomes.	Combine virtual and traditional training techniques for future pharmacy training. Enhance interactivity by converting pre-recorded videos into live sessions. Address communication gaps and technical support issues to improve virtual learning experiences.

**Bawadi et al. (2023) [33]**	Qatar	Health professions (43 students and 14 clinical instructors)	Qualitative study	Shift to virtual internships (VIs) using online platforms with case discussions, virtual projects, and clinical knowledge delivery.	Online Platform	Focus group discussions (students) and semi-structured interviews (faculty).	Skills navigation, stressors, technical issues, quality of learning, professional identity formation.	Major challenges: lack of skills for navigating VIs, professional and social stressors, limited clinical experience, technical issues, and lack of communication/feedback. Students expressed concerns about missed practical opportunities, inadequate real-life experience, and lack of preparedness to meet training goals. Professional identity development was hindered by limited clinical practice and insufficient mentorship.	Introduce innovative technologies and simulation-based teaching to support clinical education. Provide mentorship programs for students. Further studies are needed to assess the long-term impact of virtual internships on professional identity development.

**Benomar et al. (2024) [53]**	Morocco	Pharmacy (176 students)	CSS	E-learning during the pandemic using videoconferencing and digital tools	Online Platform	Google Forms questionnaire (19 questions)	E-learning satisfaction, frequency of use, challenges, and preferred teaching methods.	72.7% were satisfied with e-learning, but only 43.2% believed it positively impacted academic performance. Main issues included poor teaching quality (47%), distractions (32%), and lack of interaction. 97% rejected e-learning for practical work.	Introduce interactive elements like simulations and virtual laboratories for practical courses. Improve network connectivity and teaching quality. Combine e-learning with face-to-face education for blended learning.
**Elberkawi et al. (2022) [54]**	Libya	University instructors & undergraduate students (216 students and 101 instructors)	CSS	E-learning during COVID-19 pandemic using online platforms and digital tools.	E-learning	Two separate questionnaires for students and instructors with Likert-scale responses.	Social issues, accessibility issues, instructor-related issues, and student-related issues.	Both students and instructors agreed on the necessity of face-to-face interaction for learning. Top challenges: poor internet connectivity, infrastructure limitations, and	Provide training for both instructors and students on e-learning tools. Improve infrastructure, including reliable internet and power. Use blended learning

							power shortages. Students reported anxiety during e-learning exams, while instructors faced burdens from the e-learning process. Traditional education was seen as more effective for certain subjects.	for subjects unsuitable for full e-learning. Support students to manage anxiety during online assessments.
**Elnour et al. (2023) [35]**	UAE	Pharmacy (230 students)	CSS	Off-campus e-learning with platforms such as Microsoft Teams and Moodle during the COVID-19 pandemic.	Various Online Teaching & Online Platform	A validated and piloted self-administered survey covering four domains: preparedness, attitude, experiences, and barriers/facilitators (34 statements).	Preparedness for e-learning, attitudes towards e-learning, experiences with e-learning, and barriers/facilitators.	Students experienced challenges such as lack of motivation, concentration difficulties, and internet connectivity issues. Majority preferred on-campus learning due to better interaction and communication. Negative effects	Improve preparedness for e-learning through training and infrastructure enhancements. Blend practical and on-campus training with online learning. Address mental health concerns and student motivation during e-learning. Conduct further

							on mental and physical health (e.g., stress, lethargy, back pain). Despite challenges, students acknowledged facilitators like technical support and recorded lectures. E-learning did not fully equip students with practical and clinical skills for future pharmacy practice.	research on innovative technologies like AI to enhance pharmacy education.
**Elsalem et al. (2020) [45]**	Jordan	Undergraduate Medical Sciences (1019 students)	CSS	Remote e-exams implemented during the COVID-19 pandemic lockdown	E-learning	Online survey with 29 questions covering stress, contributing factors, and behavioral changes.	Stress levels, factors contributing to stress, and behavioral changes (diet, sleep, physical activity, smoking habits).	32% reported increased stress with remote e-exams. Main stress factors: exam duration (78%), navigation issues (76%), technical problems (60%),	Develop robust and reliable e-exam platforms. Conduct mock e-exams to prepare students. Provide stress management and awareness programs

							and exam environment. Behavioral changes included increased consumption of caffeine, energy drinks, fast food, and reduced physical activity and sleep hours. Females and non-medicine students reported higher stress levels.	addressing dietary habits, physical activity, and mental health during e-learning periods.
**Elsalem et al. (2021) [44]**	Jordan	Undergraduate faculties of Medicine, Dentistry, Pharmacy, Nursing, and Applied Medical Sciences (730 students)	CSS	Remote electronic exams (E-exams) conducted during COVID-19 using various online assessment methods	E-learning	A 16-question survey exploring preferences, academic dishonesty, and potential exam improvements.	Preference for remote E-exams vs. in-campus exams, exam-related stress, exam dishonesty, and measures to reduce misconduct.	68.2% of students preferred in-campus exams over remote E-exams. Major challenges included increased time/effort for preparation, inappropriate exam questions, and technical	Enhance remote exam platforms with proctoring tools and improved question design. Consider blended assessments combining quizzes, reports, and exams. Address dishonesty

								issues. 40% reported academic dishonesty during remote exams. Factors influencing preference: academic major, GPA, and appropriateness of questions. Measures suggested to reduce dishonesty included online proctoring, substituting exams with other assessments, and limiting exam durations.	through structural improvements and alternative assessment methods.
**Halat et al. (2022) [37]**	Lebanon	Bachelor of Pharmacy (BPharm) students and faculty	Descriptive Study	Shift from face-to-face to remote learning using synchronous and asynchronous modes, with	Various Online Teaching	Faculty feedback survey and grade trend comparisons.	Instructional changes, online engagement, faculty satisfaction, assessment	Emergency transition to online teaching due to the economic crisis and COVID-19	Implement blended learning with modern ICT tools for future program delivery. Enhance faculty

		(Sample size not reported)		adaptations in laboratory courses, experiential education, and assessments.			modifications, and grade trends.	pandemic. Assessment methods adapted to include quizzes, assignments, projects, and open-book exams. Faculty reported challenges with student engagement, exam transparency, and technical issues. Grades deviated initially during the crisis but returned to near-normal levels by Spring 2020/21.	training on virtual pedagogies and online assessment methods. Introduce workshops for improving student engagement and maintain the quality of practical education.
**Jan et al. (2022) [21]**	Saudi Arabia	Pharmacy (172 students)	CSS	Online learning via virtual platforms during the COVID-19 pandemic.	Various Online Teaching & Online Platform	Web-based questionnaire assessing knowledge, attitude, practices (KAP), and	COVID-19 knowledge, preventive practices, attitude towards learning, and e-learning tools	Students demonstrated good knowledge of COVID-19 (mean knowledge score: 3.16 ± 1.11 out of 5). 77.3% of students always	Strengthen student access to reliable internet and e-learning platforms. Enhance virtual teaching tools and support

					impact on education		wore masks, but only 43% regularly sanitized when shopping. COVID-19 significantly impacted the learning process for 71.7% of students. 60.5% believed virtual classes were effective, with females showing higher satisfaction. Internet access (53.5%) and computer skills (51.7%) were moderate challenges for students.	faculty training. Promote awareness campaigns for preventive measures to combat future health crises.
**Jarab et al. (2022) [46]**	Jordan	Pharmacy and PharmD Undergraduates and	CSS	Distance learning via online platforms implemented during the	Online Teaching	Custom-designed questionnaire with three main factors:	Students' satisfaction levels, impact on academic	Distance education had a negative impact on students' satisfaction,	Improve distance learning systems to enhance interactivity and technical

		postgraduate (880 students)		COVID-19 pandemic		negative impact, positive impact, and general impact.	performance, and daily routines.	particularly regarding the quality of education and clinical training. Gender, nationality, university type, and field of study significantly influenced satisfaction levels. Students expressed dissatisfaction with reduced clinical exposure and direct interaction.	infrastructure. Implement strategies for better clinical training through virtual simulations or hybrid approaches. Address factors such as internet reliability and student engagement.
**Kim et al. (2021) [31]**	Saudi Arabia	Undergraduate Pharmacy (19 students)	Descriptive Study	Two-week online course covering clinical and scientific topics, primarily focused on diabetes and drug-drug interactions, integrating	Online Platform	Post-course survey and course outcomes (student presentations, group projects)	Student satisfaction, engagement, and learning outcomes	High satisfaction with course organization, projects, and overall value (survey results averaged 92–97%). Interactive tools like group discussions and	Encourage social interaction early to improve group work. Use blended learning approaches in the future for international programs. Expand online

				problem-solving and group work.				competitions enhanced learning. Challenges included managing group dynamics in a virtual international setting.	international teaching opportunities beyond the pandemic.
**Mohammad and AlMuhaissen (2023) [47]**	Jordan	Pharmacy (24 students)	CSS	Online laboratory sessions replaced hands-on labs during the COVID-19 pandemic	Online Platform	Microsoft Forms-based questionnaire with 21 Likert-scale items.	Practical skills, communication skills, and student preferences for online versus face-to-face learning	73% of students agreed that online learning negatively affected their practical skills. 76% believed face-to-face lab sessions improved their communication skills. Third-year students faced more difficulties using lab equipment and glassware than senior students. 69% of students	Integrate blended learning approaches where theoretical components are taught online, but practical skills are delivered face-to-face. Offer additional face-to-face sessions to compensate for missed hands-on experience during online learning. Tailor curricula to address differences in student

							did not prefer online labs compared to face-to-face sessions. Students felt online labs lacked hands-on experience and failed to deliver essential practical skills.	experiences based on their academic year.
**Mohammed et al. (2023) [52]**	Iraq	Pharmacy (278 students)	CSS	E-learning during COVID-19 pandemic using online platforms.	Online Teaching	A structured questionnaire using Likert scales and multiple-choice questions.	Students' preferences for delivery modes, perceived benefits, challenges, and attitudes towards e-learning.	42.44% preferred face-to-face lectures over online sessions. 72.66% highlighted the advantage of studying at home, while 74.82% reported a lack of patient connection as the main drawback. Preferred topics for online lectures: pharmacoeconomics (83.09%),	Address technical and infrastructure challenges to improve e-learning. Implement blended learning models to balance theoretical content delivery online and practical aspects face-to-face. Ensure active learning engagement to optimize the

								medicinal chemistry (77.69%), and pharmacology (71.22%). Technical issues (62.23%) and reduced interactivity were significant challenges. Face-to-face teaching was perceived as more effective for knowledge acquisition (48.56%) and clinical skills development (39.92%).	benefits of remote learning.
**Panda et al. (2021) [29]**	Saudi Arabia	Pharmacy students & teachers (148 students & 46 faculty members)	CSS	Online teaching due to COVID-19 using platforms like Zoom, Google Meet, Cisco WebEx, and Blackboard.	Online Teaching	Online questionnaire with multiple-choice and open-ended questions	Effectiveness of online teaching, constraints, and preferences for online versus face-to-face learning	54.9% of students and 39.1% of faculty cited internet connectivity as the main constraint. Students favored flexibility,	Address internet connectivity issues and technical challenges. Include animated and video content for better understanding

								revisiting recorded content, and fewer distractions. Faculty valued punctuality, attendance, and technical improvements. 63% of faculty and 54.2% of students found online teaching "effective" but not superior to face-to-face teaching. Majority believed traditional face-to-face classes could not be completely replaced.	and retention. Conduct frequent formative and summative assessments to improve engagement and learning outcomes.
**Rabbani et al. (2021) [36]**	UAE	Final-year Bachelor of Pharmacy (52 students & 7 preceptors)	Descriptive Study	Virtual experiential training for community pharmacy and hospital	Online Teaching	Feedback survey with closed and open-ended questions (students) and	Perceived effectiveness, challenges, and learning outcomes of virtual training.	79.2% of students found virtual rotations useful and convenient. 89.6% believed the training	Develop an integrated model combining virtual tools with traditional experiential

			placements during the COVID-19 pandemic.		focus group discussions (preceptors).		simulated real clinical settings effectively despite limitations. Challenges included lack of patient encounters, increased screen time, and technical issues. Preceptors acknowledged the need for improved planning and greater student engagement.	methods. Include live virtual patient encounters and interprofessional activities. Enhance technical infrastructure and provide mental health support for students.
**Sabry et al. (2020) [38]**	Egypt	Final-year pharmacy (220 students)	Descriptive Study	Students designed and distributed an online survey to evaluate the Egyptian population's knowledge, attitude, and practices	Online teaching	Online surveys designed and distributed by students, followed by data analysis and reporting	Knowledge, attitude, and practice (KAP) related to COVID-19 among the Egyptian population.	26,000 responses were collected through student-distributed surveys. Students analyzed data, prepared mini-manuscripts, and presented their findings. Students reported positive	Use innovative online tools for student assessments and experiential learning. Address technical challenges like internet connectivity and provide

				regarding COVID-19.				experiences, particularly gaining insights into COVID-19 and practical research skills such as data analysis and interpretation.	additional training for faculty and students to adapt to online methodologies.
**Saeed and Almendeel (2023) [30]**	Saudi Arabia	Pharmacy (2030 students)	CSS	Online learning during the COVID-19 pandemic using synchronous and asynchronous methods.	Online Teaching	Self-administered questionnaire divided into demographic details, perceptions, and barriers (21 questions).	Students' satisfaction, perceived effectiveness, and barriers to online learning.	87.5% praised the effectiveness of online learning. 85% were satisfied and supported continuing e-learning post-pandemic. 59% reported poor internet connection as the primary access barrier. 56.2% reported discomfort with online education as a personal barrier. Female students and	Improve internet access and technical infrastructure. Address barriers faced by early-year students through support and guidance. Integrate e-learning with traditional face-to-face teaching for optimal outcomes.

								senior students (5th year) showed higher satisfaction compared to males and junior students. Students appreciated the flexibility of recorded lectures and found online learning improved their independence and technological skills.	
**Salama and Altaif (2022) [48]**	Jordan	Pharmacy (230 students)	CSS	Online learning during the COVID-19 pandemic using a blended approach	Online Teaching	Structured questionnaire using Google Forms, with a focus on challenges, preferences, and experiences	Preferences for teaching methods, challenges in online learning, and student perceptions of online education	75.2% preferred a blended learning method (online + face-to-face), while only 11.8% preferred fully online learning. Key challenges reported included: Communication issues (87%)	Encourage blended learning approaches that combine online and face-to-face education. Improve communication channels and student assessment strategies.

								Student assessment (65%) Use of technology tools (35%) Time management difficulties (48%). A small percentage of students (8%) reported stress and anxiety related to online learning. 83% of students were willing to incorporate online expertise into future practices.	Provide better support for time management and technological skill development.
**Shawaqfeh et al. (2020) [19]**	Saudi Arabia	Pharmacy (309 students)	CSS	Online learning during COVID-19 pandemic using online platforms	Online teaching	3-domain survey questionnaire focused on preparedness, attitude and barriers	preparedness, attitude and barriers	Results indicated that about 61.4% of the students agreed that college of pharmacy was well-prepared and ready for the online education	The need for training for students and faculty was highly associated with the preparedness and barriers domains

	during the emerging COVID-19 pandemic with complete transition into online education. The results also indicated that 49.2% of the students showed positive attitude toward the provided online learning. The results indicated that about 34% of the students identify some barriers toward the provided online learning. There were strong association between the need for training on how to receive online courses and preparedness	rather than the infrastructure or computer literacy, so the school can improve their experience by addressing these needs.

			and barriers scores.	

[1] Abbreviations: MMS: Mixed-method study, CSS: Cross-sectional study, DC: Description Correlation.
